# ACCEPTABILITY AND PRELIMINARY EFFICACY OF THE TAKING CARE OF US INTERVENTION FOR COUPLES LIVING WITH HEART FAILURE

**DOI:** 10.1093/geroni/igad104.1321

**Published:** 2023-12-21

**Authors:** Karen Lyons, Carol Whitlatch, Amanda Vest, Jenica Upshaw, Stacy Hutton Johnson, Anna Walters, Christopher Lee

**Affiliations:** Boston College, Boston, Massachusetts, United States; Benjamin Rose Institute on Aging, Cleveland, Ohio, United States; Tufts University School of Medicine, Boston, Massachusetts, United States; Tufts University School of Medicine, Boston, Massachusetts, United States; Canopy Consultants Inc., Arlington, Massachusetts, United States; Boston College, Boston, Massachusetts, United States; Boston College, Boston, Massachusetts, United States

## Abstract

There are over 1 million hospital admissions and 3 million emergency room visits for heart failure (HF) annually in the USA. Despite significant impact of HF on both members of the care dyad, there are few interventions focused on optimizing the mental and relational health of the dyad. The Taking Care of Us© (TCU) intervention is a communication-based program for the couple living with HF. Sessions focus on facilitating greater shared appraisal, communication, collaborative illness management, confidence, and support between partners. Thirty seven couples were randomized to TCU (18 couples) or an educational control (19 couples). Both programs were delivered virtually over two months. Adults with HF (AwHF) were, on average, 69.9 (SD=9.6) years old and predominantly male (70%). Eighty one percent of couples participated in at least one session (attrition was relatively even across conditions and primarily due to poor health and partner drop-out). Twenty-four couples (65%) completed follow-up surveys. The majority of TCU participants were satisfied with the program (AwHF 82%; care partners 100%) and felt they understood their partner’s needs better (AwHF 91%; care partners 91%). TCU care partners felt the relationship had improved (91%) and felt more confident managing HF (91%). Depressive symptoms decreased in TCU AwHF (ES=.60) compared to the active control group. TCU couples also reported increases in collaboration (ES=.50-.70) and communication (ES=.35-.50). Discussion will focus on the individual versus dyadic benefits of the TCU program, recruitment and retention challenges, and qualitative feedback from participants regarding strengths of the program and areas for improvement.

